# Monitoring of high-risk children in health services: A geospatial mixed-methods study

**DOI:** 10.1590/1518-8345.5806.3777

**Published:** 2023-01-06

**Authors:** Bianca Machado Cruz Shibukawa, Roberta Tognollo Borota Uema, Natan Nascimento de Oliveira, Rosana Rosseto de Oliveira, José Luis Guedes dos Santos, Ieda Harumi Higarashi

**Affiliations:** 1 Universidade Estadual de Maringá, Maringá, PR, Brazil.; 2 Universidade Federal de Santa Catarina, Florianópolis, SC, Brazil.

**Keywords:** Child Health, Patient Dropouts, National Health Programs, Program Evaluation, Maternal-Child Health Services, Ambulatory Care, Saúde da Criança, Pacientes Desistentes do Tratamento, Programas Nacionais de Saúde, Avaliação de Programas e Projetos de Saúde, Serviços de Saúde Materno-Infantil, Assistência Ambulatorial, Salud Infantil, Pacientes Desistentes del Tratamiento, Programas Nacionales de Salud, Evaluación de Programas y Proyectos de Salud, Servicios de Salud Materno Infantil, Atención Ambulatoria

## Abstract

**Objective:**

to analyze adherence, non-adherence and abandonment of the monitoring of children referred to the high-risk reference centers of *Rede Mãe Paranaense*.

**Method:**

a parallel and convergent mixed-methods study, in which both approaches have the same weight. The study loci were two high-risk outpatient services from the South of the country. In the quantitative part, 3,107 medical charts of high-risk children were analyzed and the spatial distribution was performed. In the qualitative part, interviews were conducted with 29 health professionals, in addition to 34 family members, and content analysis was performed. Two databases were produced, which were analyzed separately and eventually integrated.

**Results:**

the rates regarding adherence to monitoring are decreasing, mainly in the municipalities that are far away from the high-risk outpatient services, and the non-adherence and abandonment rates are increasing. In the reports by the representatives and the manager, a failure was observed between the transportation offer and the active search flow of the absent patients, which contributes to the increase in the non-adherence and abandonment rates and to the consequent decrease in adherence.

**Conclusion:**

in high-risk children, adherence is decreasing and the non-adherence and abandonment rates increased.

## Resumo

**Objetivo:**

analisar a adesão, não adesão e abandono do acompanhamento das crianças encaminhadas aos centros de referência de alto risco da Rede Mãe Paranaense.

**Método:**

estudo de método misto paralelo convergente, no qual ambas as abordagens possuem o mesmo peso. O cenário do estudo foram dois ambulatórios de alto risco do sul do país. Na parte quantitativa, analisou-se 3.107 prontuários de crianças de alto risco e realizou-se distribuição espacial. Na parte qualitativa, realizou-se entrevistas com 29 profissionais de saúde, além de 34 familiares e se procedeu com a análise de conteúdo. Houve a produção de dois bancos de dados analisados separadamente e com integração ao final.

**Resultados:**

as taxas de adesão ao acompanhamento estão diminuindo, principalmente nos municípios distantes dos ambulatórios de alto risco, e as taxas de não adesão e abandono estão em ascensão. Observou-se nos relatos dos representantes e do gestor, falha entre a oferta de transporte e o fluxo de busca ativa dos pacientes faltosos, o que contribui para o aumento das taxas de não adesão e abandono, e a consequente diminuição da adesão.

**Conclusão:**

a adesão das crianças de alto risco está em decréscimo ao mesmo tempo em que as taxas de não adesão e abandono aumentaram.

## Resumen

**Objetivo:**

analizar la adherencia, no adherencia y el abandono del seguimiento de niños derivados a los centros de referencia de alto riesgo de la *Rede Mãe Paranaense*.

**Método:**

estudio del método convergente paralelo mixto, en el que ambos enfoques tienen el mismo peso. El escenario de estudio fueron dos consultorios externos de alto riesgo del sur del país. En la parte cuantitativa se analizaron 3.107 historias clínicas de niños de alto riesgo y se realizó una distribución espacial. En la parte cualitativa, se realizaron entrevistas a 29 profesionales de la salud y a 34 familiares, y se realizó el análisis de contenido. Se crearon dos bases de datos que se analizaron por separado y se integraron al final.

**Resultados:**

las tasas de adherencia al seguimiento están disminuyendo, fundamentalmente en los municipios alejados de los consultorios externos de alto riesgo, y las tasas de no adherencia y abandono van en aumento. Se observó en los relatos de los representantes y del gestor, un problema entre la oferta de transporte y el flujo de búsqueda activa de pacientes ausentes, lo que contribuye al aumento de las tasas de no adherencia y abandono, y, por ende, a la disminución de la adherencia.

**Conclusión:**

la adherencia de los niños de alto riesgo está disminuyendo y, a su vez, aumentan las tasas de no adherencia y abandono.

**Highlights**

(1) There is a trend to a reduction in adherence to the appointments.

(2) A failure in the transportation offer was identified in the active search flow.

(3) After almost 10 years since the network was created, there are still weaknesses attracting the population.

(4) Increase in the rates regarding non-adherence and abandonment of the monitoring of high-risk children.

## Introduction

A general effort by several countries around the world is observed, as well as coping means that assist in infant mortality, justifying the creation of specific health programs aimed at maternal and child monitoring that seek better results for the families and children^1^.

In this sense, *Rede Cegonha* (RC), was instituted in 2011 by the Federal Government with the objective of reducing maternal and child mortality, based on improving the care provided to the dyad^2^. And *Rede Mãe Paranaense* (RMP), based on RC, was initiated in the state of Paraná in 2012, focused on guided functionality in risk stratification of pregnant women and children, a reference and counter-reference system. Stratification of the groups both during the pre- and post-natal periods, such as child monitoring in the first year of life, takes place in three classes (low, intermediate and high)^3^.

At high risk, children are classified according to the following characteristics: perinatal asphyxia, hyperbilirubinemia with exsanguinotransfusion, delayed neuropsychomotor development, prematurity, low birth weight, genetic diseases, malformations, positive neonatal screening, vertical transmission diseases and severe malnutrition^3^.

By means of the high-risk stratification, the Program points to the need for periodic monitoring during the first year of life in the reference outpatient service^3^. However, a number of studies signal cases of families that fail to attend the appointments and even regarding discontinuity itself in the health monitoring services for high-risk children. Non-adherence to monitoring occurs when the first appointment is missed. In turn, monitoring discontinuity is characterized as treatment abandonment^4^ ^-^ ^5^.

It is known that children’s attendance to the appointments is influenced by intrinsic and extrinsic factors. Among the intrinsic factors connected to the mothers, it is perceived that adherence is influenced by the meanings they attribute to follow-up of the care provided to the child; the appointments refer to situations of diseases and personal demands or related to domestic obligations^6^. In relation to the extrinsic factors, adherence can be closely linked to the waiting time for the appointment, to the availability of scheduling in health services and to the very stance of the health professionals^7^ ^-^ ^8^. Previous studies have already evidenced that the farther the distance to the consultation locus, the lower the chances for the population to receive proper care in the maternal and child health services^9^ ^-^ ^10^.

To advance in the investigation of this phenomenon, a research approach is necessary that includes both geographical factors as well as the perception of family members, health professionals and managers. Thus, conducting a mixed-methods study can contribute to a broader understanding of the problem researched.

It is noted that effectiveness of the child monitoring programs is inherent to the family members’ adherence during the entire follow-up period recommended. Reflection on the conditions that go beyond the adherence, non-adherence and abandonment process experienced by the families and their children, as well as among health professionals, is essential to ensure proper functioning of the programs. Therefore, the objective defined was to analyze adherence, non-adherence and abandonment of the monitoring of children referred to the high-risk reference centers of *Rede Mãe Paranaense*.

## Method

### Study design

A geospatial, parallel and convergent mixed-methods study in which the same weight is assigned to the quantitative (QUAN) and the qualitative (QUAL) components of the research^11^. When conducting the study, the methodological rigor criteria for mixed-studies were followed, according to the *Mixed-Methods Appraisal Tool* (MMAT)^12^.

### Study locus

The study loci were two high-risk reference centers of RMP, references for the care of a Health Region of the state of Paraná, located in the Brazilian South region, added to the 30 municipalities that make up the region.

### Period

Data collection took place between December 2019 and March 2021.

### Population

For the quantitative phase of the study, 3,107 medical charts of children referred and monitored by the high-risk outpatient services were selected. In turn, in the qualitative phase, the population consisted of a health manager responsible for RMP in the study region, 28 health professionals representing the municipalities, eight family members from the group that did not adhere to child monitoring, 11 family members from the follow-up abandonment group and 15 family members of from group that completed the care schedule.

### Selection criteria

For the selection of medical charts, the records of all children stratified as high risk, referred to the RMP reference centers from January 2015 to December 2019 and monitored until December 2020 or who abandoned the follow-up were considered eligible. After reading them, the medical charts were divided into three groups based on the Guiding Line^3^: adherence, non-adherence, and abandonment.

The adherence group was defined as those children that initiated and finished their planned monitoring period. The non-adherence group consisted in children referred for monitoring, but who never attended the appointment in the reference service. The abandonment group was characterized by children who initiated their monitoring but whose family members failed to attend the appointments before finishing it.

The inclusion criteria in the family groups were as follows: being responsible for taking the child to the appointments, and fitting into one of the three study groups (adherence, non-adherence or abandonment) in the last six months prior to the initial collection date, in order to preserve memory/recall of the facts.

For selection of the managers, each municipality was asked to appoint a representative, according to the following inclusion criteria: participating in the meetings of the RMP leading group, in which the network strategies are discussed and aligned. It was understood that the participants were sufficiently engaged in the initiative and able to indicate practicalities and difficulties encountered in their respective municipalities.

The following criteria were listed for the selection of employees responsible for RMP within the Health Region headquarters: having at least one year of experience in the network and being responsible for monitoring the leading group and RMP.

### Study variables

The quantitative variables of the study were as follows: adherence, non-adherence and abandonment rates, calculated based on the proportion between the number of cases in each stratum and the population of high-risk live births, multiplied by 1,000. 15% of the number of births was calculated as the population of high-risk live births^13^.

The qualitative data sources were the recorded reports by the managers, health professionals and family members.

### Instruments used for data collection

For collecting quantitative data, a structured questionnaire was used, consisting of the name of the children’s municipalities of origin, outcome (adherence, non-adherence and abandonment) and number of the medical chart.

For qualitative data collection, a semi-structured script was elaborated for each of the groups of interviewees: health managers, health professional responsible for RMP, and adherence, non-adherence and abandonment family groups. For the family members, the questions were about the reasons for referral to high risk and the reasons that led the family members to finish, abandon or not adhere to the follow-up. For the Health Region managers and representative, the questions were intended to investigate the process of child monitoring, active search for absentees and provision of support to family members to facilitate adherence to the program.

### Data collection

In the quantitative phase, electronic access to the medical charts was granted by signing a responsibility form in both institutions. A list of children referred to the services during the period of interest was generated, thus enabling initiation and verification of the children’s follow-up in the high-risk outpatient services. The data of interest were collected in an instrument prepared by the researcher herself. The diverse information referring to the number of live births was obtained by means of queries in the public platform of the Live Birth Information System (*Sistema de Informação sobre Nascidos Vivos*, SINASC).

In the qualitative phase, semi-structured interviews were conducted remotely, due to the COVID-19 pandemic scenario. It was considered unsuccessful when there was no response in the contacts made with the family during seven days of the week at different times.

On the agreed day and time, the researcher made the call and read the free and informed consent form. After accepting to participate in the research, the interview was conducted following the script for each family group. The telephone call was recorded and later transcribed in full.

The “abandonment group” consisted in 30 families, although 13 refused to participate in the study; in five cases, contact was unsuccessful; and the number/contact provided was nonexistent in the case of four families. A total of eight interviews were conducted in this family subgroup. The “non-adherence” family group consisted of 35 eligible families. However, the number provided was nonexistent for 17 and contacts were unsuccessful in seven cases. Eventually, 11 family members were approached. In the “adherence group”, of the 33 families approached, 15 had provided nonexistent telephone numbers and contacts were unsuccessful in three situations, which resulted in 15 family members interviewed.

For data collection with the group of RMP health managers and collaborators, an indication list was provided with the names and telephone numbers of a representative from each municipality. From this list, contact was made with the person indicated, scheduling a time according to the professional’s availability. At the time of the invitation, it was asked if the person was familiar with the Google Meet platform and the professional email address was requested so that a formal invitation to the interview was sent. Before starting the interview, the free and informed consent form was read, also requesting authorization to record the interview.

The data exhaustion criterion was adopted for ending data collection. In other words, all the previously scheduled interviews were conducted, even if the data had already presented theoretical saturation.

### Data treatment and analysis

For the quantitative part, the spatial distribution of the adherence, non-adherence and abandonment rates by year and during the five-year period (2015-2019) contemplated by the study was performed. Elaboration of the caption was based on the interquartile values, namely: first, second and third quartiles, and maximum value. The value of zero was included as a separate category, not being considered for calculation of the interquartile categories. The rates and spatial distribution were calculated with the aid of the Quantum Gis (QGIS) 3.10 software.

In turn, in the qualitative part, the data were transcribed with the support of Microsoft Word 2019^®^. After transcription, the data were exported to the NVivo Release 1.5.1 software and analyzed based on the content analysis stages^14^. In the pre-analysis, the data were systematically explored according to the principles of completeness, representativeness, homogeneity and pertinence. The initial coding phase was performed to give rise to conceptions about adherence, non-adherence and abandonment of the monitoring of the children referred to the RMP high-risk reference centers. The main topics were identified and named through an approximation and distancing process. Finally, with the results generated, charts were prepared to illustrate the findings, which were later submitted to inference and interpretation from the perspective of the national and international literature.

As this is a study of the convergent parallel mixed-methods type, there was production and independent analysis of two databases, the results of which were integrated at the end, in order to identify convergences and divergences. Combination of the data from the QUANT and QUAL results occurred at the end of the analysis of each method, from the elaboration of a spatial distribution map with the synthesis of the qualitative findings. The assumptions of the *Rede Mãe Paranaense* Program^3^ were adopted for analysis and interpretation of the results.

### Ethical aspects

The ethical and legal precepts were observed, respecting Resolution 466/2012 of the National Health Council. To ensure anonymity of the subjects interviewed, their reports were identified by the letters G, referring to the managers (“*gerentes*” in Portuguese”), P for the health professionals, and F for the family members, followed by the Arabic number corresponding to the interview. The study was approved by the Research Ethics Committee, with CAAE: 24906719.9.0000.0104.

## Results

In the quantitative phase, 3,107 medical charts of children from both high-risk outpatient services responsible for meeting the demand of all 30 municipalities of the health region approached were analyzed. Of these, 17.4% (540) did not adhere to RMP, 26.7% (829) finished child monitoring as recommended by RMP, and 55.9% (1.738) abandoned the high-risk follow-up.

**Figure 1:**
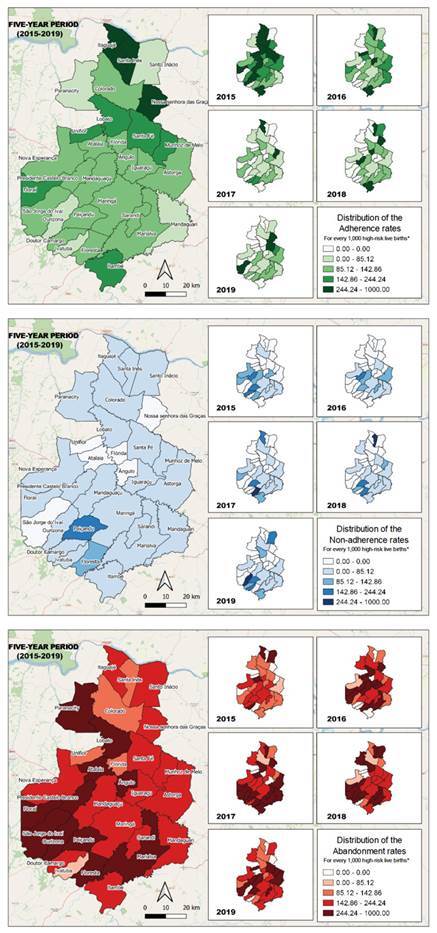
Spatial distribution of the adherence, non-adherence and abandonment rates by year and five-year period. Paraná, Brazil, 2021

^*^To calculate the adherence, non-adherence and abandonment rates, we used the estimate of the population of high-risk live births, which was obtained considering 15% of all live births^13^

Adherence to child follow-up in the high-risk outpatient services according to the RMP recommendation is decreasing. The spatial distribution of the rates shows that, increasingly fewer children have completed the corresponding health monitoring. A phenomenon that draws the attention is that, despite the decrease in adherence, the municipalities with the highest adherence rates continue to be those with the largest distances from the consultation sites (Figure 1).

The families with children stratified as high-risk and that decide not to undergo child monitoring (non-adherence group) present well-distributed rates across all 30 municipalities. However, an increase in this behavior was identified in the spatial distribution of the non-adherence rates, mainly in cities surrounding the municipality where the high-risk outpatient services are located (Figure 1).

The abandonment rates are variable across the municipalities, but the cases with higher incidence of follow-up abandonment were in the cities around the Health Region headquarters. The maps allow seeing the evolution of these rates, represented with darker colors over the years (Figure 1).

In relation to the qualitative data, 28 representatives from the municipalities were interviewed: 26 (92.8%) nurses, one (3.6%) nutritionist and one (3.6%) social worker. Regarding the role performed, 20 (71.4%) were health coordinators, seven (25%) were Primary Care or Family Health Strategy nurses and one (3.6%) was a social worker. The representative from the Health Region was a nurse, who was the head of the Maternal and Child Health division.

The adherence family group consisted of 15 participants: 12 (80%) mothers and three (20%) fathers. 12 (80%) had a partner and three (20%) did not. Of these, 10 (66.7%) had Complete High School, three (20%) had Higher Education and two (13.3%), Elementary School. In this group there were family members who lived in the city where the outpatient services operated and also others living in municipalities more than 100 km away. The main reasons to refer these children to the high-risk outpatient services were extreme prematurity (6; 40%) and congenital malformation (3; 20%).

The non-adherence family group consisted of eight participants, where all the interviewees were mothers, 50% (4) had a partner and 50% (4) did not. Of these, 62.5% (5) had Complete High School, 6.66% (1) had Elementary School and 13.33% (2), Higher Education. In this group there were family members that lived in the same city of the outpatient services, as well as in municipalities more than 15 km away. The main reason to refer these children to the high-risk outpatient services was toxoplasmosis (2; 13.33%) and 3 (20%) ignored the reason for monitoring.

The abandonment family group consisted of 11 participants: 9 (81.81%) mothers, one (9.09%) father and one (9.09) grandmother. In relation to partners, 7 (67.64%) had a partner and four (36.36%) did not. Six (54.54%) had Complete Elementary School, four (36.36%) had High School level and one (9.09%), Higher Education. In this group there were family members who lived in the city where the outpatient services operated and also from municipalities more than 75 km away. The main reasons to refer these children to the high-risk outpatient services were prematurity in four (36.36%) cases and malformation in two (20%).

Two categories emerged in content analysis: Reasons for adherence, non-adherence or abandonment of child health monitoring; and Primary Care and Health Region strategies for continuity of high-risk child follow-up. Each of them is described below.

### Reasons for adherence, non-adherence or abandonment of child health monitoring

The reasons for adherence evidenced in the family group are related to feelings of fear and concern, while at the same time they express a strong commitment to maintenance of the child’s well-being and health (Figure 2).

In the statements of the non-adherence family group, the reasons for not complying with the monitoring refer to the impression that the child is apparently healthy and, therefore, does not need care, to the use of health insurance, to the lack of support to adolescent mothers evidenced in the report that they could not leave school to take the child to the appointment, added to the lack of understanding of the importance of child monitoring and the suppression of information provided by the health team, in favor of information without scientific evidence.

In the abandonment cases, the interviewees emphasized the distance between the consultation places and their homes, the long waiting times, lack of information about the need to monitor the child and the intrinsic feeling that the child was well and healthy, in addition to fear of contamination by the COVID-19 virus.

**Figure 2:** Statements by the family members from the groups corresponding to adherence, non-adherence and abandonment of child monitoring of *Rede Mãe Paranaense*. Paraná, Brazil, 2021

Adherence	Non-adherence	Abandonment
*Fear and concern: [...] he had cardiac arrest in the second month of life, then I never gave up taking him to every appointment, I didn’t want to lose my son. F10*	*Healthy child: I’ve raised nine children, apart from my brothers, I grew up here, I know when we need to go to the doctor. We need to go to the doctor when we’re sick [...] a healthy child doesn’t need to. F04*	*Distance: It’s just that I live in Marialva, right, so I took it, but they didn’t say anything else and I didn’t take it anymore. F08 We moved to another city, [...] I think it’s 50 minutes from Maringá. I don’t have my own car and with the pandemic I don’t have the courage to go with health transportation. F04*
	*Health plan: I started working a little before discovering the pregnancy, so I had no right to earn them in the health insurance because of absence of a plan, [...] I did all the prenatal care in private services. F05*	*Waiting time: The waiting time [...] We arrived very early, six in the morning, [...] I went very early because I needed to go back to work, so I could leave at almost one in the afternoon. F11*
*Commitment to health: Knowing the evolution in the issue of his prematurity, how he was, how growth was evolving, the feeding issue. F01 Concern even for knowing if everything was OK, because it was a very complicated pregnancy, [...] concern even to know if she was really OK. F03 In addition to my daughter being special, I have a commitment to her as a father, as a human being, [...]. F14*	*Lack of support: I won’t miss school to take him to the appointment, my mother and father work. I won’t stop my life because of the child [...]. F03*	*Fear of contamination: I was terrified of the pandemic and thought, “I’m not going out with him”, he was fine thank God, they were guiding well and I ended up choosing not to take him. F09 Because of the pandemic, it wasn’t a matter of not being able to go, it was due to the pandemic. Afraid that we’ll end up contaminating the baby itself. F05*
	*Lack of understanding: They told me a lot of things, but I searched on Google and saw that it was more to instill fear on me, which it is rare to happen, [...] now I have Internet and I discover things, it’s no use wanting to scare me. F02*	

### Primary Care and Health Region strategies for continuity of the high-risk child follow-up

Faced with the behavior of adherence, non-adherence and abandonment to high-risk child monitoring, the representatives of the municipalities and the health region reported the development of strategies that assist the families in the beginning and maintenance of the services proposed by RMP. However, this situation lacks better organizational conditions. Two assistance modalities were identified for maintenance of child health monitoring: transportation and active search, as shown in Figure 3:

**Figure 3:** Statements by the representatives from the municipalities and the Health Region. Paraná, Brazil, 2021

Representatives from the municipalities	Representative from the Health Region
Transportation	
*The municipality makes transportation available for the person to be able to go, to be able to return. P26*	*The network did provide resources for sanitary transportation, but already embedded in the transfer. So within the ordinance it states that we have to invest in sanitary transportation, but there’s no specific resource for this, it’s within the resource that will pass on funds to the municipalities, within Rede Cegonha in the whole thing. G1*
*In the unit itself, the transportation voucher is available [...] then you look for the social worker and she delivers quietly. P16*	
*We have scarce public transportation, to take us at the time of the appointment, now there’s only a van to pick us up. Then it depends a lot on when the last one leaves to take us back. P13*	
*There is no such thing [...] it’s the mother with her own resources that goes there. P27*	
*Our support here as an aid, not asking the mother to go demanding, but in fact support is more difficult. P21*	
**Active search**	
*When there’s a shortage, if the high-risk people don’t warn, we don’t know that he missed the appointment. If they do warn, we contact them, we go to the house, see what happened. P5*	*The active search has to be done by Primary Care because it is them who are directly linked to this user [...] Many of the municipalities don’t have a consolidated or adequate FHS team for the population [...] We always pick on the municipalities, because it’s the basis of everything, for all networks. So, active search has to be a part of this, but I think that over these 10 years it’s still difficult, and the difficulty of being able to carry this out at the very end, on the spot, by the managers, by the entire health team, it hasn’t been easy. G1*
*The active search is via WhatsApp, a lot of WhatsApp, mainly now during the pandemic, or even home visits. P9*	
*Unfortunately, this is one of my failures, it may be because I don’t have the city after me, but I don’t actively search for high-risk children. P26*	
*We receive an email telling us that the child missed the appointment, then we already [...] make the visits, see what happened, why she missed it, we try to understand why she did not go to the outpatient service. P23*	

Regarding the active search for the children who missed the monitoring appointments, it was evidenced that some representatives from the municipalities report that they do so and have used technology in their favor, using both email and *WhatsApp* messages to ask for the reason for not attending the outpatient services and question the families. Although this strategy is extremely feasible, some professionals reported that they do not do so or that the information did not reached them.

### Combination of the quantitative and qualitative results

**Figure 4:**
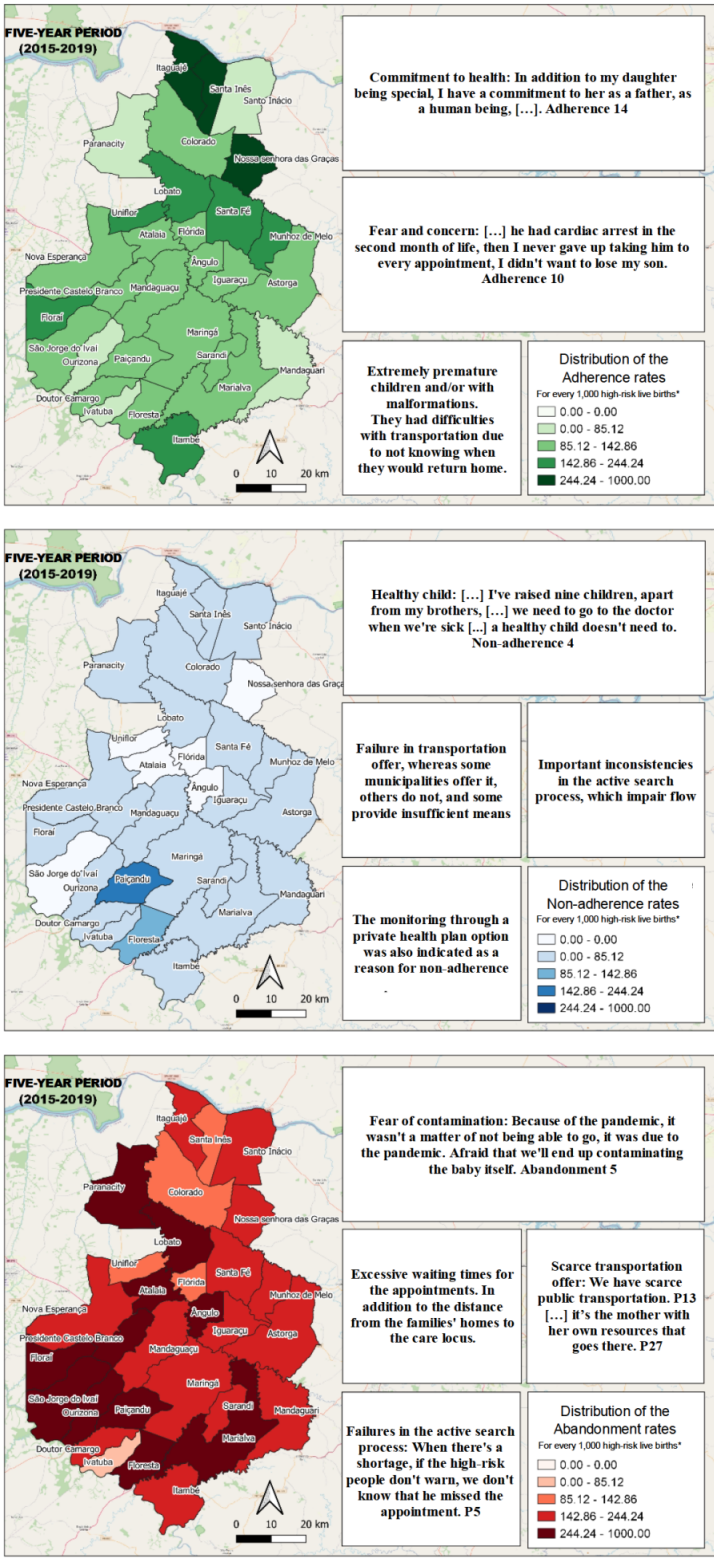
Integration of the quantitative and qualitative results about child monitoring adherence, non-adherence and abandonment. Paraná, Brazil, 2021

The combination of the quantitative (QUANT) and qualitative (QUAL) approaches by integrating the results enables recognizing convergences and divergences between the research data. Some examples of this study are presented below; data integration is also illustrated in Figure 4.

In the distribution map corresponding to the adherence rates, a movement of decreased adherence to the appointments can be seen, mainly in the municipalities that are more distant from the high-risk outpatient services. Although the rates have decreased, in the adherence group it was noticed that approximately 60% of the children treated had extreme premature0 births, or that there were malformations, a fact that can sensitize and even worry the family more and make it remain assiduous to health monitoring. In the reports made by the representatives and even by the manager, it was observed that, in some municipalities, transportation does not have a fixed time for the return trip, a fact that also hinders organization of the routine of the family and the child, consequently reducing adherence.

In the distribution map corresponding to the non-adherence rates, it can be seen that there is an increase in the municipalities that are farther away from the outpatient service headquarters and also some darker points near the headquarters, evidencing that these municipalities are also moving towards a non-adherence behavior. The family members’ reports point out that the child is relatively well and therefore does not need to be treated justify asserting that they consider the effort of a trip to be monitored in a routine appointment unnecessary. Added to this is the failure between the transportation offer and the deficiency of the active search flow of patients who miss the appointments, which contributes to the increase in the non-adherence rates.

In relation to the abandonment rates, it was noticed that they were maintained in some municipalities, although a slight decrease was evidenced. It was expected that, with the implementation of RMP, such rates would suffer a significant decline and even disappear; however, converging the map with the family members’ discourse, it was evidenced that many of them abandoned the monitoring due to the distance from the outpatient service to their home. However, it is noted that even some of the municipalities surrounding the outpatient services do not offer transportation to their patients, increasing the abandonment chances. In the reports made by the representatives from the municipalities, we also see that the active search process remains inadequate, a fact that contributed to the permanence of the abandonment rates evidenced on the map.

## Discussion

The results and, later, convergence of the QUANT phase with the QUAL phase, show that the high-risk child monitoring in health services presents some flaws, even with the performance of RMP in the state of Paraná. The network’s objective is to ensure that the patient is properly cared for since pregnancy and that she can circulate through all the care levels in an organized way. Such care does not end with birth of the child, but rather when it is monitored in a fixed, responsible and continuous manner, in order to remedy its needs and identify potential situations that may put it at risk early in time^13^.

In the last five years, there has been an increase in the rates related to the absence of child monitoring, especially in cities close to the host municipality. This context can be explained by the qualitative findings, when the family members report that the child is relatively well and that it therefore does not need to be followed-up, except in situations of illness, and many already have private health insurance and choose not to resort to the care services provided in the public network.

Added to this are situations related to the lack of a support network, as in the case of adolescent mothers, which can justify the fact that, even living in a city close to the outpatient service, it is impossible to reconcile their activities, including school life, to take the child to the appointments. Also in this context, we also have families who end up solving their doubts on the Internet and believe that this search is enough to affirm whether or not the child needs care^15^ ^-^ ^16^.

The quantitative data showed a decrease in adherence to child monitoring services, that is, despite the RMP efforts, these rates continue to decrease. However, it is noted that, even with the decrease in the care flow, the most distant municipalities are those with the highest adherence rates, in disagreement with previous studies^9^ ^-^ ^10^.

This finding can be explained by the qualitative data. The family members interviewed in the adherence group reported fear and concern about the health status of the child. Distance was not mentioned in any statement, suggesting that the commitment to the child’s health can overcome geographical barriers.

Regarding abandonment of services, the integration between the quantitative and qualitative data showed that the abandonment rates in the region under study are variable, with higher rates in cities close to the health region headquarters. From the interviews, it can be seen that the distance between the home and the health service, the difficulty to access to transportation means, the long waiting times and the COVID-19 pandemic are the main factors related to abandonment of the monitoring.

The fear related to a possibility of contamination linked to the need to use sanitary transportation to reach the outpatient services was evidenced. This fact corroborates the sensation of being unprotected when leaving the house with the child during the pandemic for routine appointments, and turns monitoring into something secondary. Although transportation is provided for in RMP, as stated by the manager, the family members’ testimonies show that the situation has not yet been adequately resolved, culminating in high abandonment rates.

Not attending the appointments is configured as a situation of higher risk, as the children had characteristics that the stratification itself already allocated as a need for special monitoring^3^. It was noticed in the family members’ reports that lack of transportation directly contributed to non-attendance to the services. This demand should be met by the management of the network itself, as the difficulty reaching the health services is already something foreseen by the program. At the same time, it is up to the representatives from the municipalities and the Health Region manager to understand the real difficulties that limit access to the health services and to delimit improvement strategies.

There are gaps between what is advocated by law and what is contained in the regulation of the network services, in particular with regard to transportation services. The difficulty in locomotion, the lack of structure and even the low articulation between the services can hinder access to health institutions, especially those of low and medium complexity. Cases in which families need to travel long distances, or live in more distant places or in neighboring municipalities, are situations that corroborate the low child care flow, characterizing situations of non-adherence and/or abandonment^17^.

Another perceived problem was lack of communication in the services regarding the active search for those who were absent and strategies to attract professionals for this purpose. While in the manager’s speech it is evidenced that such monitoring happens and must be carried out, even with the difficulties, at the same time it was inferred in the reports made by the representatives from the municipalities that many are unaware about such situation and even admit that this is a failure in the exercise of their work.

It is already stated in the literature that ineffective communication, especially in a health care network, impairs care and monitoring of users. Especially in the pediatric population, this gap causes children not to be adequately monitored and this exerts directly impacts on their growth and development. At the same time, it is important to emphasize that this lack of search for the patients who miss the appointments should not be understood by the services as a frequent routine, but should be intended to generate concern and, consequently, action. When this reality becomes incorporated into the routine and starts to be considered normal, control of its own care population is lost and what was recommended begins to be unfeasible^18^.

The culture change regarding the myth that a healthy child does not need monitoring is a structural and psychological barrier that needs to be overcome. In order to change this scenario, in addition to listening to the families and Primary Care health professionals in order to seek ways to demystify this situation, it is necessary that the transformation process is also encouraged by the manager himself, so that from this, the representatives from the municipalities devise action plans within their areas in order to improve and guarantee children’s and families’ access to the health services, so that the abandonment rates perceived in the quantitative part of the study can be reduced.

When a child went through a delicate situation at birth, such as premature delivery, or had some malformation, the family members showed greater concern with health care. The same was not evidenced regarding the care provided to healthy children. The concept of health promotion and disease prevention is reversed, now health is sought when a disease is already installed. It is necessary to empower the families to care for their children in a healthy context, aiming at longitudinal and quality care for their offspring in the long term^19^.

Caring for a child who went through an unforeseen situation at birth leads to a feeling of insecurity and fear, as idealized motherhood was abruptly interrupted by the high-risk condition. Such moment can lead to an intense search for health services, aiming at minimizing the problems and preventing potential alert situations with a potential for worsening^20^.

A dilemma evidenced by means of the data integration lies in being able to attract and raise awareness in the families of the children who evolved in a more peaceful way and already expected in the first months of life that remaining healthy and with the appointments up to date is a protective factor, and that they should not abandon child follow-up. It is noted that children with chronic conditions are already vulnerable; therefore, being part of the monitoring non-adherence and/or abandonment group makes this population more likely to present health problems in addition to needing highly complex care^21^.

Integration of the study results shows that there is much to be done in the field of child health. Implementation of the network in 2012 had the objective of ensuring comprehensive care and adequate access; however, the adherence rates presented a drop, while the non-adherence and abandonment rates are on the rise. It is understood that the pandemic can hinder the situation; however, analyzing the five-year period, it is evidenced that the lack of routine appointments was already a reality. In addition, several difficulties are evidenced in the testimonies to maintain child monitoring continuity.

As a study limitation, it is pointed out that only one Health Region was analyzed. Therefore, the data cannot be generalized at the state level. Despite that, the study supports the formulation of new policies and coping strategies aimed at improving access to child health services.

## Conclusion

Adherence of the children referred to high-risk monitoring is decreasing and the non-adherence and abandonment rates have increased. The family members consider that such results are related to distance from the health services, to moving to another city and to the belief that a healthy child does not need monitoring. Among the managers and municipal representatives, difficulties were identified in the provision of transportation assistance and in the active search for children and family members who miss the appointments.

It is believed that the study can support changes in the child care scenario. As the factors that directly contribute to adherence, non-adherence and abandonment of the monitoring appointments are known, it is possible to propose changes aimed at remedying such difficulties and improving the rates related to child health monitoring.
